# Anlotinib Downregulates RGC32 Which Provoked by Bevacizumab

**DOI:** 10.3389/fonc.2022.875888

**Published:** 2022-05-18

**Authors:** Zhujun Liu, Tingting Qin, Xiaohan Yuan, Jie Yang, Wei Shi, Xiaoling Zhang, Yanan Jia, Shaochuan Liu, Jing Wang, Kai Li

**Affiliations:** ^1^ National Clinical Research Center for Cancer, Tianjin Medical University Cancer Institute and Hospital, Tianjin, China; ^2^ Key Laboratory of Cancer Prevention and Therapy, Tianjin, China; ^3^ Tianjin’s Clinical Research Center for Cancer, Tianjin, China; ^4^ Department of Thoracic Oncology, Tianjin Lung Cancer Center, Tianjin Cancer Institute and Hospital, Tianjin Medical University, Tianjin, China; ^5^ Department of Oncology, The First Affiliated Hospital of Xinxiang Medical University, Xinxiang, China; ^6^ Hematology Center, Beijing Key Laboratory of Pediatric Hematology Oncology, Beijing, China; ^7^ National Key Discipline of Pediatrics (Capital Medical University), Beijing, China; ^8^ Key Laboratory of Major Diseases in Children, Ministry of Education, Beijing, China; ^9^ Beijing Children's Hospital, Capital Medical University, National Center for Children's Health, Beijing, China; ^10^ Research and Development Department, Jiangsu Chia-Tai Tian Qing Pharmaceutical Co., Ltd., Nanjing, China

**Keywords:** anlotinib abrogated bevacizumab resistance, anlotinib, bevacizumab, MMP2, N-cadherin, RGC32, lung adenocarcinoma, TGFβ

## Abstract

**Background:**

Bevacizumab is the representative drug in antiangiogenic therapy for lung cancer. However, it induced resistance in some neoplasm. Anlotinib, a novel multi-target tyrosine kinase inhibitor which has an inhibitory action on both angiogenesis and malignancy, is possible to reverse the resistance.

**Methods:**

Transwell migration and invasion experiments of bevacizumab with or without anlotinib were conducted to verify the activated/inhibited ability of lung adenocarcinoma cells. We sequenced A549 cells with enhanced migration and invasion abilities after bevacizumab treatment, screened out the differentially expressed gene and further confirmed by western blot and q-PCR assays. We also investigated immunohistochemical staining of tumor tissue in mice and human lung adenocarcinoma.

**Results:**

Bevacizumab facilitated migration and invasion of lung adenocarcinoma cells. Differentially expressed gene RGC32 was screened out. Bevacizumab upregulated the expression of RGC32, N-cadherin, and MMP2 through ERK-MAPK and PI3K-AKT pathways. Anlotinib downregulated their expression and reversed the effect of bevacizumab on A549 cells. *In vivo* experiments confirmed that higher-dose bevacizumab facilitated metastasis in tumor-bearing nude mice and upregulated the expression of RGC32, N-cadherin, and MMP2, whereas anlotinib abrogated its effect. Expression of both RGC32 and N-cadherin positively correlated with lymph node metastasis and stage in lung adenocarcinoma was found. Survival analysis revealed that higher expressions of RGC32 and N-cadherin were associated with poor progression-free survival and overall survival.

**Conclusions:**

Bevacizumab may promote invasion and metastasis of lung adenocarcinoma cells by upregulating RGC32 through ERK-MAPK and PI3K-AKT pathways to promote epithelial–mesenchymal transition, whereas anlotinib reverses the effect. RGC32 and N-cadherin are independent prognostic factors in lung adenocarcinoma.

## Introduction

Non-small-cell lung carcinoma is the leading cause of cancer-related deaths worldwide (1–3), and adenocarcinoma is the most common subtype with increasing yearly incidence (1–3). Antiangiogenesis therapy is an important strategy for lung adenocarcinoma (LADC) treatment. Bevacizumab, a recombinant humanized monoclonal antibody against vascular endothelial growth factor (VEGF), is a widely utilized anti-angiogenesis drug that blocks the binding of VEGF and its receptor (VEGFR), then inhibits accordant downstream signals (4); its clinical efficacy is highly recognized (5, 6).

However, some clinical trials have revealed that bevacizumab only improves the progression-free survival (PFS) rather than overall survival (OS) in some cancers (7, 8). Moreover, recent studies have found that it promotes the invasion of glioma cells (9–12), glioblastoma, and colorectal cancer which are characterized by highly infiltrative and other malignant behavior (13, 14). Therefore, it is important to elucidate the underlying mechanisms to avoid the adverse effect of bevacizumab.

Tumor neovascularization is mainly mediated by three signal pathways: the VEGF/VEGFR, fibroblast growth factor (FGF)/fibroblast growth factor receptor (FGFR), and platelet-derived growth factor (PDGF)/platelet-derived growth factor receptor (PDGFR). Bevacizumab can only inhibit VEGF/VEGFR, while FGF/FGFR (15, 16) and transforming growth factor-β (TGFβ) are upregulated during the treatment process (17), which is the probable reason for resistance to bevacizumab. Anlotinib is a multi-target inhibitor that can inhibit VEGFR, FGFR, and PDGFR (18, 19). Our previous studies have shown that anlotinib can also inhibit the upregulation of TGFβ caused by bevacizumab (17). Therefore, in theory, anlotinib may reverse resistance, which is important in the clinical practice of lung cancer treatment.

However, it is currently unclear which molecules downstream of FGFR and TGFβ are involved in the resistance to bevacizumab and impacted by anlotinib. Therefore, the aim of this study was to confirm the effect of bevacizumab on TGFβ signaling, to elucidate downstream signaling molecules, and to determine whether anlotinib can reverse resistance to bevacizumab.

## Material and Methods

### Patients and Tumor Tissue Samples

A total of 121 specimens from patients with LADC, 20 adjacent tissue specimens, and 11 normal lung tissue specimens were collected from patients who underwent surgery at Tianjin Medical University Cancer Institute and Hospital, Tianjin, China between December 2011, and November 2015. All the specimens were independently confirmed by two experienced pathologists. Each patient signed an informed consent form prior to participation. All patients completed the follow-up and met the criterion of not receiving chemotherapy before surgery. This study was conducted in accordance with the recommendations of the Tianjin Medical University Cancer Institute and Hospital Ethics Committee. The Ethics Committee of Tianjin Medical University Cancer Institute and Hospital (Tianjin, China) approved the use of human tissues in this study. Paraffin-embedded tissues were provided by the Department of Pathology of the hospital. The clinical and pathological data of the patients were recorded in detail. Lung cancer staging was performed according to the latest National Comprehensive Cancer Network guidelines.

### Reagents

Immunohistochemical antibodies for RGC32, MMP2, and N-cadherin were purchased from Abcam (Cambridge, UK). Western blot antibody for RGC32 was purchased from USBiological (Swampscott, MA, USA). Primary antibodies to P-AKT, AKT, P-ERK, ERK, β-actin, HRP-conjugated secondary antibodies, and inhibitors of U0126 and LY294002 were purchased from Cell Signaling Technology (Danvers, MA, USA). The P-SMAD2 (S465/S467) TR-FRET Cell Signaling Assay Kit was purchased from Bioauxilium (Montreal, Canada). TGFβ1 was purchased from Sino Biological (Beijing, China). Bevacizumab was purchased from Roche (S20120068, Basel, Switzerland). Anlotinib was provided by Jiangsu Chia-Tai Tian Qing Pharmaceutical Co. Ltd.

### Cell Culture

The human LADC cell lines NCI-H1299 (EGFR^wild type^), NCI-H358 (EGFR^wild type^), HCC-827 (EGFR^19del^ mutation), NCI-H1650 (EGFR^19del^ mutation), NCI-H3255 (EGFR^L858R^mutation), NCI-H1975 (EGFR^L858R,T790M^ mutation), NCI-H460 (EGFR^wild type^), and A549 (EGFR^wild type^) were purchased from the American Type Culture Collection (Manassas, VA, USA). A549-EGFP cells were generated in our laboratory. Cell culture was performed as described in our previous study (20).

### Cell Migration and Invasion

Migration and invasion assays were performed using a transwell filter (8-μm pore size; Corning, USA). Transwell membranes were coated with a Matrigel matrix (1:7 dilution; Corning) for invasion assays. A549 and NCI-H1299 cells were treated with bevacizumab, anlotinib, or both for 24 h and then seeded onto the top precoated chamber (5×10^4^ cells per well for 16-h migration evaluation and 1×10^5^ cells per well for 24-h invasion evaluation) in 200 μL FBS-free RPMI 1640 medium (Gibco, USA). The RPMI 1640 medium (600 μL) containing 20% FBS was placed in the bottom chamber. After 24 h of incubation at 37°C in 5% CO_2_, the inserts were rinsed, and the lower surface of the membrane with migrating or invasive cells was fixed with 4% paraformaldehyde for 30 min and then stained with 0.1% crystal violet (Solarbio, China). The number of cells that migrated per field was counted in five randomly chosen fields per well at 200× magnification (ECLIPSE Ti, Nikon, Tokyo, Japan).

### Cell Viability Analysis

Cell viability analysis was performed as previously described (20).

### RNA-Seq

RNA-seq and bioinformatic analyses were performed according to our previous study (21).

### Quantitative Real-Time Polymerase Chain Reaction Gene Expression Analysis

Samples were collected and homogenized in TRIzol reagent (Invitrogen, USA). RNA extraction and reverse transcription were performed according to the standard procedures. For PCR amplification of the cDNA fragment coding for the targeted genes, the sense and antisense primer sequences for human RGC32 and GAPDH were as follows: 5′′-TGCCAGAGGGGACAAGAC-3′′ and 5′′-GCAAGCAGGTAAACAAAGTCAG-3′′; 5′′-TCCATGACAACTTTGGTATCG-3′′ and 5′′-TGTAGCCAAATTCGTTGTCA-3′′. Relative mRNA expression levels were calculated after normalization to those of the internal control (GAPDH).

### Western Blot Analysis

Western blot analysis was performed as previously described (20).

### ELISA

Conditioned media samples from cells treated with bevacizumab and anlotinib were collected. VEGFA and TGFβ1 concentrations were determined using a human VEGFA and TGFβ1 ELISA Kit (R&D Systems). The absorbance was measured using an EpochTM microplate reader (BioTek, Winooski, VT, USA).

### Homogeneous Time Resolved Fluorescence

A549 cells were treated with TGFβ1 (1 ng/mL for 1 h), and then with anlotinib (5000-4.9 nM for 1, 2, 4 h). The cells were collected to determine the Phospho-SMAD2 (S465/S467) concentration. The Phospho-SMAD2 (S465/S467) TR-FRET cell signaling assay was performed according to the manufacturer’s instructions (Bioauxilium).

### Animals and Subcutaneous Lung Cancer Model

Female nude mice (BALB/c-nu, 4 w old) were purchased from the Model Animal Center of Nanjing University and housed in a pathogen-free animal facility with ad libitum access to water and food. This study was conducted in accordance with the principles of the Basel Declaration and the recommendations of the Institutional Animal Care and Research Advisory Committee of Tianjin Medical University. The study protocol was approved by the committee. To induce a subcutaneous xenograft lung cancer model, we subcutaneously injected nude mice with 5×10^6^ A549-EGFP cells into the right shoulder. Tumor formation was observed after 7 d.

These mice were divided into three groups: a vehicle group, a 10 mg/kg bevacizumab group based on the preclinical animal experimental data (the instruction of bevacizumab), and a 50 mg/kg bevacizumab group (the dose used in our previous study) (17) in order to observe possible different effects at low and high doses. Bevacizumab was administered 7 d after tumor cell injection *via* intraperitoneal injection twice a week for 8 w. The vehicle group received homologous human IgG control. Metastases were imaged using an IVIS Spectrum Imaging System (Caliper Life Science, Hopkinton, MA, USA), and A549-EGFP tumors could be clearly visualized. Although autofluorescent signals were provided by naïve mice, high-intensity fluorescent signals from tumors were distinguishable and after normalization over the background signal.

To study the antitumor effect of anlotinib, we divided the mice into four groups: vehicle, anlotinib, bevacizumab, and combined. Bevacizumab was administered in the same manner as described above for 3 w. Anlotinib was administered daily for 3 w by gavage.

Tumor volume and body weight were measured twice per week. Tumor size was measured using a dial caliper in a blinded manner. Tumor volumes were determined using the following equation volume = width^2^×length/2. The mice were euthanized at the end of the experiment and the tumors were collected and weighed. Some of the tumor samples from each mouse were fixed in 4% paraformaldehyde solution, and the remaining samples were stored at -80°C.

### Immunohistochemical Assay

Immunohistochemical staining was performed as previously described (22).

### Statistical Analysis

A two-tailed test was used for all statistical analyses. Statistical significance was set at P<0.05. Data are expressed as the mean ± SD. *In vitro* experimental data were examined using ANOVA followed by unpaired Student’s t-tests. χ2 and Fisher’s exact tests (accurate probabilistic method) were used to analyze the differential expression of RGC32, N-cadherin, and MMP2 in various tissues, and clinical pathological parameters. The correlation between the positive rate of RGC32 and the positive rate of N-cadherin expression was analyzed using Spearman correlation analysis. Survival curves were calculated using the Kaplan–Meier method, and differences were estimated using the log-rank test.

## Results

### Bevacizumab Promotes Migration and Invasion of LADC Cells

**Figure 1:**
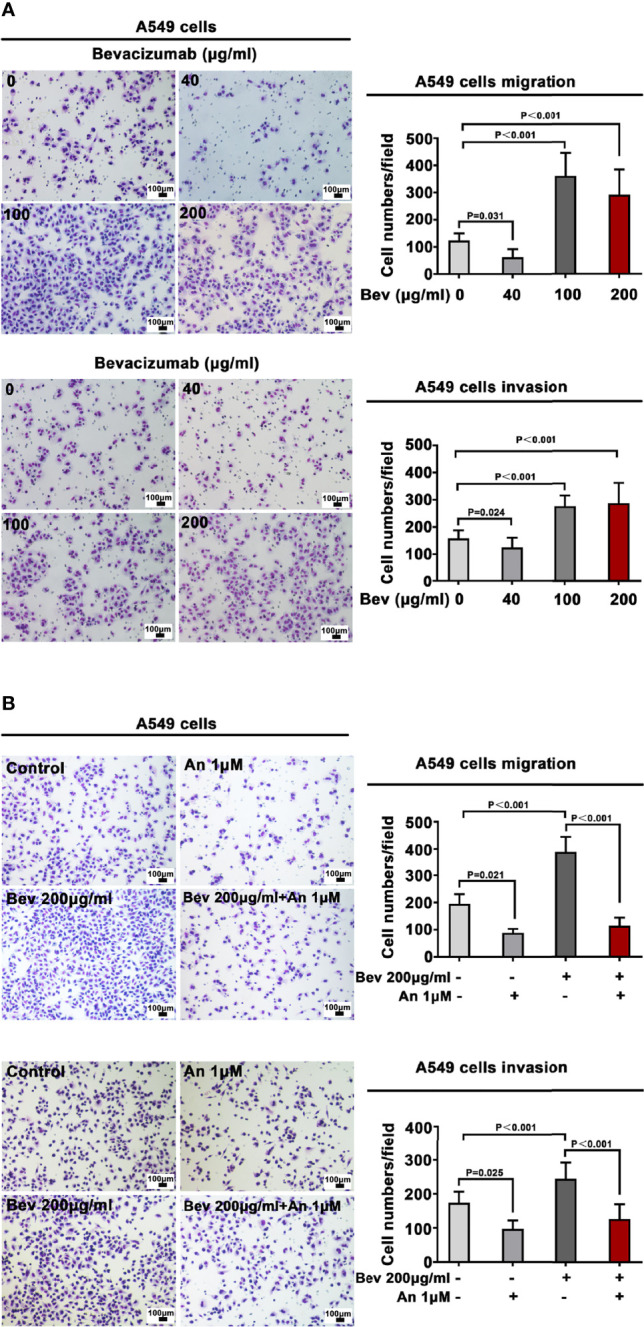
Bevacizumab promotes A549 cell migration and invasion. **(A)** Migration and invasion of A549 cells under different concentrations of bevacizumab. **(B)** Migration and invasion of A549 cells treated with 200 μg/mL bevacizumab, 1 μM anlotinib, or both. Representative images are shown. Magnification ×200. n=3. Bev: bevacizumab.

A549 and NCI-H1299 cells were treated with bevacizumab at 40, 100, and 200 μg/mL for 24 h to evaluate the various influence of different concentrations on migration and invasion. We found that 100 and 200 μg/mL bevacizumab promoted the migration and invasion of A549 (**Figure 1A**) and NCI-H1299 cells (**Figure S1**), whereas its lower concentration did not.

We also observed an effect of bevacizumab on cell proliferation, which showed that bevacizumab had minimal effect on A549 and H1299 cell proliferation (**Figure S2**).

### Anlotinib Alone or Combined With Bevacizumab Inhibits Migration and Invasion of A549 Cells

To define a suitable effective concentration of anlotinib, cell viability was evaluated using the MTT assay after LADC cell lines were exposed to anlotinib for 24 h. The IC50 of anlotinib treatment was 9.495 μM in A549 cells (**Figure S3A**) and 12.25 μM in NCI-H1299 cells (**Figure S3B**). To evaluate the influence of anlotinib on the migration and invasion of A549 cells, we treated A549 cells with 1 μM (much less than the IC50 in order that enough live cells would remain for observation regarding its bio-function) anlotinib alone or combined with 200 μg/mL bevacizumab for 24 h. Transwell analysis showed that anlotinib alone depressed the migration and invasion of A549 cells, whereas 1μM anlotinib combined with 200 μg/mL bevacizumab abrogated the effect of bevacizumab on the migration and invasion of A549 cells (**Figure 1B**).

### Bevacizumab Promotes Migration and Invasion of LADC Cells Through Up Regulation on RGC32

**Figure 2:**
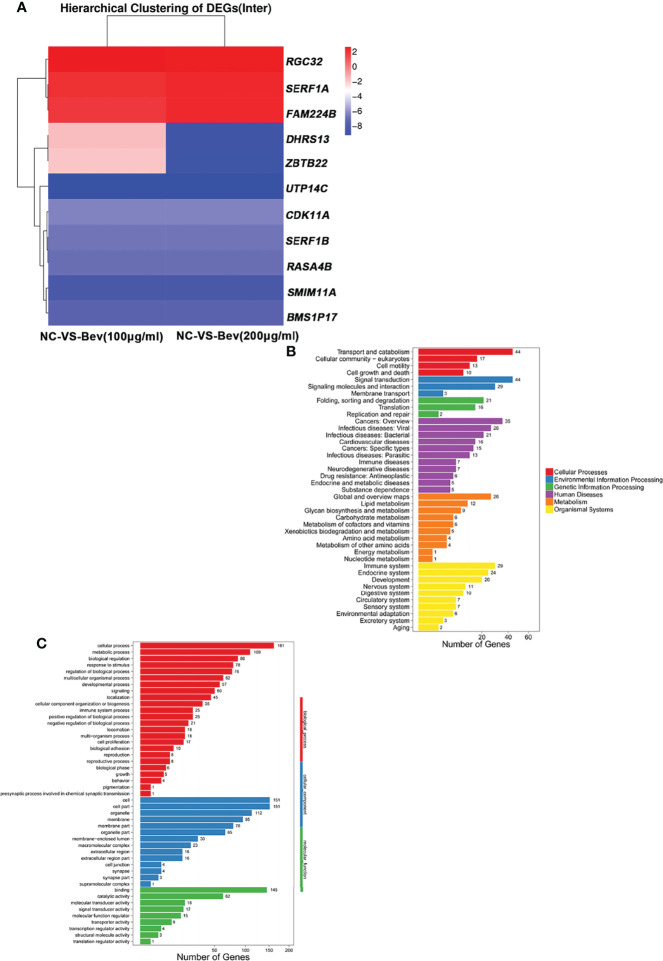
Transcriptome analysis of differentially expressed genes induced by bevacizumab. **(A)** Heat map representation of differentially expressed genes in A549 cells in the control (NC), 100 μg/mL bevacizumab-treated and 200 μg/mL bevacizumab-treated groups. Red color represents gene upregulation and blue color represents gene downregulation. **(B, C)** GO analysis and KEGG pathway enrichment analysis of the differentially expressed genes in A549 cells after bevacizumab treatment.

We explored the molecular mechanism underlying the promotion of migration and invasion of A549 cells by analyzing the differentially expressed genes (DEGs) at the transcriptome level. We screened 357 DEGs (P<0.05, log2^Fold Change^<-1 or log2^Fold Change^>1) and found 178 upregulated and 179 downregulated genes. We generated a heatmap of DEGs that shows effect of bevacizumab on A549 cells at the gene expression level (**Figure 2A**).

GO and KEGG pathway analyses of the DEGs revealed that downregulated genes (log2^Fold Change^<-1) and upregulated genes (log2^Fold Change^>1) are involved in multiple biological processes and signaling pathways (**Figures 2B, C**). Basing on relevant literature, we identified three genes possibly related to the change in tumor cell migration and invasion ability including responsible gene to complement 32 (RGC32) (**Figure 2A**).

### ERK-MAPK and PI3K-AKT Signaling Pathways are Involved in Up-Regulating RGC32 Expression Induced by Bevacizumab in A549 Cells

**Figure 3:**
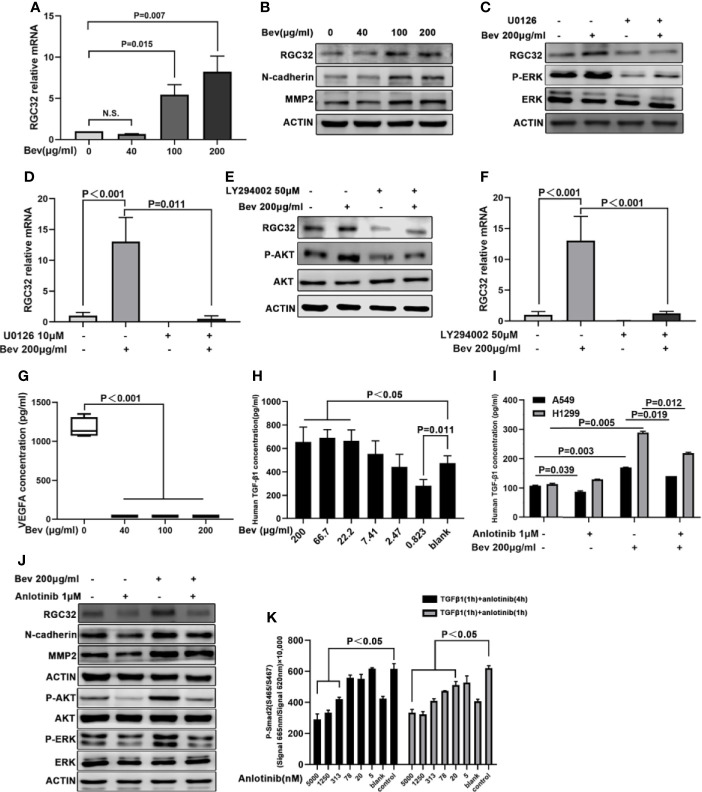
Anlotinib reverses upregulated RGC32 expression by bevacizumab *via* ERK-MAPK and PI3K-AKT signaling. **(A)** q-PCR analysis of RGC32 mRNA levels in A549 cells treated with vehicle and bevacizumab at 6-h time point. Values were calculated using the ddCT method. n.s: nonsignificant. **(B)** Western blot analysis showing RGC32, N-cadherin and MMP2 protein levels in A549 cells after bevacizumab treatment at different concentrations. **(C)** Western blot analysis of RGC32 and P-ERK protein levels in A549 cells after bevacizumab treatment in the presence or absence of MAPK inhibitor U0126. **(D)** q-PCR analysis of RGC32 mRNA in A549 cells in response to bevacizumab treatment in the presence or absence of U0126. **(E)** Western blot analysis of RGC32 and P-AKT protein levels in A459 cells following bevacizumab treatment in the presence or absence of PI3K inhibitor LY294002. **(F)** q-PCR analysis of RGC32 mRNA in A549 cells in response to bevacizumab treatment in the presence or absence of LY294002. **(G)** Cultured A549 cells treated with different concentrations of bevacizumab were assessed by ELISA to measure VEGFA. n = 3. **(H, I)** Cultured A549 cells treated with bevacizumab and anlotinib were assessed by ELISA to measure TGFβ. n = 2. **(J)** Western blot analysis of RGC32, N-cadherin, MMP2, P-AKT, and P-ERK levels in 200 μg/mL bevacizumab and 1 μM anlotinib treated A549 cells. **(K)** HTRF analysis of P-Smad2 protein levels after anlotinib treatment. n = 2. Bev, bevacizumab.

To verify the sequence analysis results, we conducted a q-PCR assay and found that 100 and 200 μg/mL bevacizumab upregulated the mRNA expression of RGC32 (**Figure 3A**). To understand the change in protein level of RGC32, we carried out western blot assays to detect the expression of RGC32, N-cadherin, and MMP2, which are related to epithelial–mesenchymal transition (EMT). The expression levels of RGC32, N-cadherin and MMP2, which are both stimulated by RGC32, were upregulated after treatment with a high concentration of bevacizumab (**Figure 3B**). To confirm the change in RGC32, we also administered 200 μg/mL bevacizumab to NCI-H1299 cells and found that RGC32 expression was remarkably increased (**Figure S4**). We also conducted western blotting to assess the baseline expression level of RGC32 in eight LADC cell lines, including A549 and NCI-H1299 (**Figure S5**). Results demonstrated that RGC32 was expressed in many LADC cell lines; the expression of NCI-H358 was the highest, followed by NCI-H1299, A549, NCI-H1975, and NCI-H460, while the expression of HCC-827, NCI-H1650, and NCI-H3255 was relatively low.

To elucidate whether the ERK-MAPK and PI3K-AKT signaling pathways are involved in RGC32 expression after bevacizumab treatment, we treated A549 cells with the MAPK inhibitor U0126 (10 μM) and the PI3K inhibitor LY294002 (50 μM). Treatment with U0126 and LY294002 reduced the upregulation of RGC32 induced by bevacizumab at both the protein and mRNA levels (**Figures 3C, D** for U0126 and **Figures 3E, F** for LY294002). Furthermore, we determined VEGFA concentration in the medium by ELISA and found that VEGFA levels after bevacizumab treatment were 30 times lower than those in the vehicle-treated group (**Figure 3G**). TGFβ1 levels were escalated after bevacizumab treatment in a dose-dependent manner (**Figure 3H**), and decreased by anlotinib (**Figure 3I**).

### Anlotinib Down-Regulates the RGC32 Expression Promoted by Bevacizumab Through the ERK-MAPK and PI3K-AKT Pathways

To determine the effect of anlotinib on RGC32, we added anlotinib and bevacizumab to A549 cells. We found that 1 μM anlotinib reversed the upregulation of RGC32, N-cadherin, and MMP2 expression induced by 200 μg/mL bevacizumab (**Figure 3J**).

To examine the effect of anlotinib on the ERK-MAPK and PI3K-AKT signaling pathways, we administered anlotinib at 0.01, 0.1, and 1 μM concentration to A549 cells. We found that anlotinib at 0.01 μM decreased P-ERK and P-AKT expression in a dose-dependent manner (**Figure 3J**).

### Anlotinib Reduces the Phosphorylation of Smad2 Protein in A549 Cells

The phosphorylation of Smad2 was significantly stimulated by 1 ng/mL TGFβ1 for 1 h in A549 cells. Anlotinib reduced the phosphorylation of Smad2 protein induced by 1 ng/mL of TGFβ1 in a dose-dependent manner (**Figure 3K**).

### Bevacizumab Promotes Metastasis of Subcutaneously Transplanted Tumors in Mice

**Figure 4:**
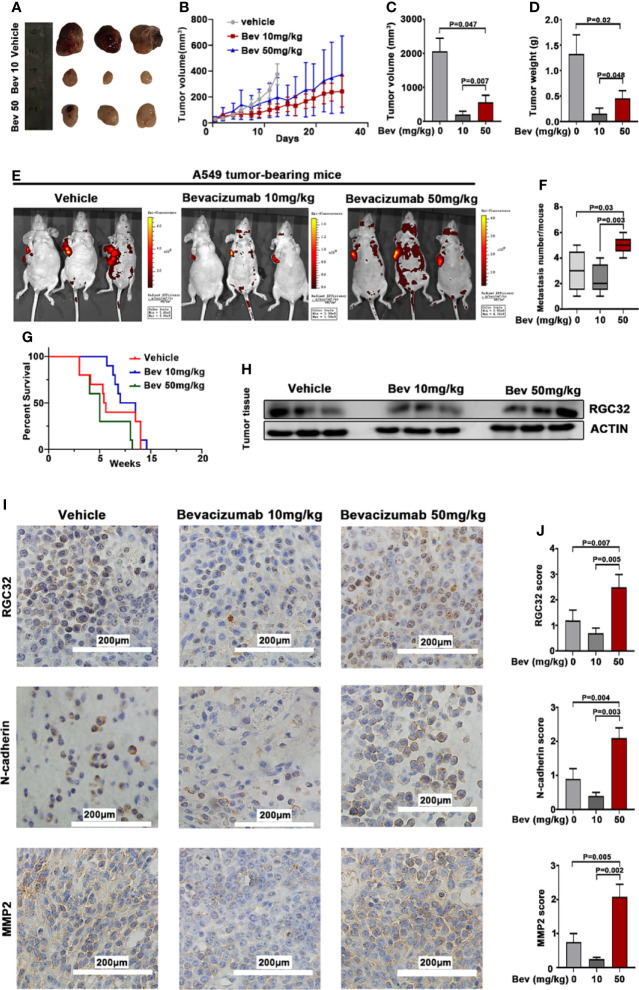
Bevacizumab elevates RGC32, N-cadherin, and MMP2 expression, and promotes tumor metastasis while inhibiting primary tumor growth in subcutaneous transplanted tumors in mice. **(A)** Typical image of the primary tumors that formed in mice grafted with A549 cells on the right shoulder, which were treated with or without bevacizumab. Ruler unit: cm. **(B)** Primary tumor growth curves for each group. **(C)** Column diagram for the tumor volume with bevacizumab treatment. n = 3. **(D)** Column diagram for the tumor weight in each group. n = 3. **(E)** Representational images of mice with tumor cells transplanted and spontaneous metastasis monitored by the IVIS spectral imaging system. Colored bars represent radiation efficiency. **(F)** Boxplot analysis of metastases focus number. n = 3. **(G)** Survival after 10 mg/kg bevacizumab, 50 mg/kg bevacizumab or vehicle treatment (n = 10, P = 0.0235) after documented tumor burden. Treatment started at week 0. Median survival 5.32 w for 50 mg/kg bevacizumab vs 7.69 w for 10 mg/kg bevacizumab treatment. **(H)** Western blot analysis of RGC32 expression in tumor tissues. **(I)** IHC to detect RGC32, N-cadherin and MMP2 in primary tumors. Representative images are shown. Magnification ×400. **(J)** Column diagram of IHC scores in each group. Each column is the average of five random fields per tumor. n = 3. Bev, bevacizumab.

To evaluate the effect of bevacizumab on metastasis *in vivo*, we established a mouse model of human lung cancer by transplanting A549-EGFP cells subcutaneously into the right shoulder of nude mice. The injection of bevacizumab inhibited tumor growth in the 10 and 50 mg/kg groups compared with the vehicle group, but the primary tumors in the 50 mg/kg group were larger than those in the 10 mg/kg group (**Figure 4A**). We observed the primary tumor size twice a week for 8 w. Bevacizumab inhibited tumor growth compared to the vehicle group, whereas the tumor growth in the 50 mg/kg bevacizumab group was remarkably higher than that in the 10 mg/kg group (**Figure 4B**). The medium tumor volume and tumor weight in the 50 mg/kg bevacizumab group were twice those in the 10 mg/kg bevacizumab group (**Figures 4C, D**). The number of metastases was detected using the IVIS Spectrum Imaging System, which demonstrated an average of 1.5 lesions per mouse in the 10 mg/kg bevacizumab group and an average of five lesions per mouse in the 50 mg/kg bevacizumab group, compared with three lesions in the vehicle group (**Figures 4E, F**). A significant survival advantage was observed in the cohort of mice that were treated with 10 mg/kg bevacizumab, with a median survival of 7.69 weeks versus 5.32 weeks in the cohort treated with 50 mg/kg bevacizumab (**Figure 4G**); log-rank test, P = 0.0235). These results indicate that high-dose bevacizumab can promote tumor metastasis by inhibiting tumor growth.

### Bevacizumab Upregulates the RGC32, N-Cadherin, and MMP2 Expression of Subcutaneously Transplanted Tumors in Mice

We conducted western blot analysis to detect RGC32 protein expression in the tumor tissues. The results showed that RGC32 protein expression increased in the 50 mg/kg bevacizumab group compared with the vehicle and 10 mg/kg bevacizumab group (**Figure 4H**).

We also conducted immunohistochemical staining in the primary tumors of the three groups to further determine the effect of bevacizumab on the expression of RGC32, N-cadherin, and MMP2. The results showed that the protein expression of RGC32, N-cadherin, and MMP2 was upregulated in the same pattern in the 50 mg/kg bevacizumab group when compared with the vehicle and 10 mg/kg bevacizumab groups (**Figures 4I, J**). These results indicate that RGC32 plays an important role in bevacizumab-induced tumor metastasis.

### Anlotinib Reverses the Effect of Bevacizumab on Subcutaneously Transplanted Tumors in Mice

**Figure 5:**
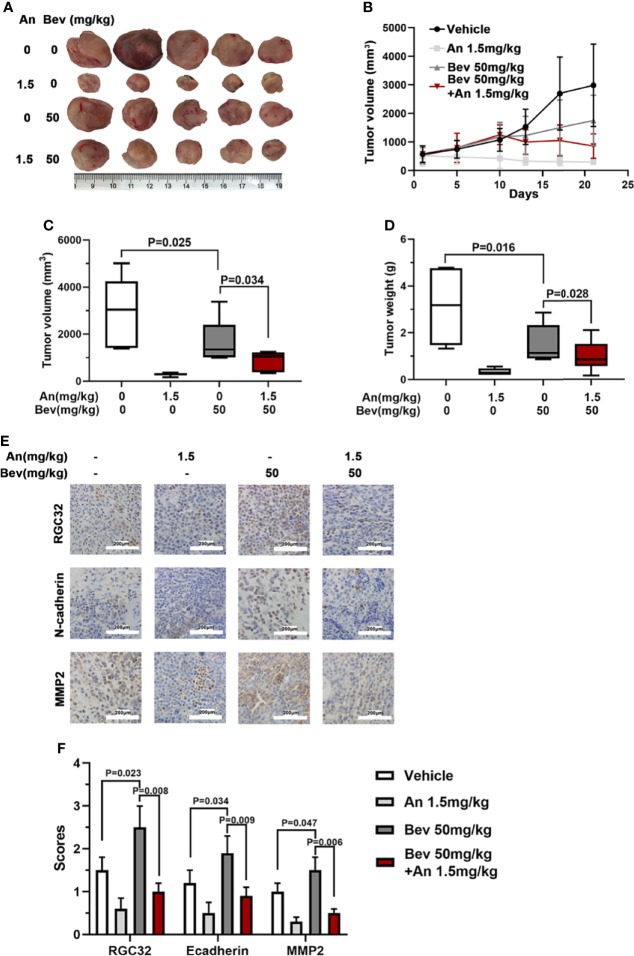
Anlotinib abolishes the effect of bevacizumab and downregulates RGC32, N-cadherin, and MMP2 expression in tumors of mice. **(A)** Typical image of the primary tumors that formed in mice grafted with A549 cells on the right shoulder, which were treated with bevacizumab, anlotinib or both. Ruler unit: cm. **(B)** Tumor growth curves for each group. **(C)** Column diagram of the tumor volume for each group. n = 5. **(D)** Column diagram of the tumor weight for each group. n = 5. **(E)** IHC to detect RGC32, N-cadherin and MMP2 in primary tumors. Representative images are shown. Magnification ×400. **(F)** Column diagram of IHC scores in each group. Each column is the average of five random fields per tumor. n = 5. Bev, bevacizumab; An, anlotinib.

We further conducted *in vivo* experiments to evaluate the effect of anlotinib on subcutaneously transplanted tumors in mice. The same animal model was used for this study. Treatment with anlotinib plus bevacizumab remarkably inhibited tumor growth compared with bevacizumab, and the group treated with anlotinib alone also showed a decreased tumor size compared with the vehicle group (**Figure 5A**). Anlotinib markedly inhibited tumor growth compared with the vehicle group, and the tumor sizes in the combined group were also smaller than those in the bevacizumab alone groups (**Figure 5B**). The median tumor volume and tumor weight in the anlotinib alone group were 10 times lower than those in the vehicle group, and the median tumor volume and tumor weight in the bevacizumab group were twice those in the combined group (**Figures 5C, D**). These results indicate not only an anti-tumor effect of anlotinib, but also the ability to reverse the enhancement caused by 50 mg/kg bevacizumab.

### Anlotinib Downregulates RGC32, N-Cadherin, and MMP2 Expression in Subcutaneously Transplanted Tumors in Mice

To further determine the effect of anlotinib on the expression of RGC32, N-cadherin, and MMP2, we performed immunohistochemical staining in the four groups. Compared with the vehicle group, the expression levels of RGC32, N-cadherin, and MMP2 in the combined group were remarkably lower than those in the bevacizumab group and anlotinib groups (**Figures 5E, F**). These results indicate that anlotinib can decrease the expression of RGC32 and probably reverse the effect of bevacizumab on tumor metastasis.

### Correlation of RGC32/N-Cadherin Expression With Clinical Pathological Parameters and Survival

The baseline clinical characteristics of 121 patients with LADC are summarized in **Table 1**. No correlation was found between the expression of RGC32 or N-cadherin and age, sex, smoking history, EGFR mutation, and KRAS mutation. However, the expression of RGC32 and N-cadherin was correlated with TNM stage and lymph node metastasis in patients. In addition, we found a positive correlation between RGC32 and N-cadherin expression (r = 0.192, P = 0.035).

**Table 1:** Baseline characteristics and expression of RGC32/N-cadherin of 121 lung adenocarcinoma patients.

Characteristics	RGC32 expression			N-cadherin expression		
	Positive, n (%)	Negative, n (%)	P- value	Positive, n (%)	Negative, n (%)	P- value
**Age**			0.925			0.909
<45	6 (6.1%)	1 (4.6%)		5 (5.8%)	2 (5.7%)	
45-59	50 (50.5%)	12 (54.5%)		43 (50.0%)	19 (54.3%)	
≥60	43 (43.4%)	9 (40.9%)		38 (44.2%)	14 (40.0%)	
**Gender**			0.495			0.929
male	53 (53.5%)	10 (45.5%)		45 (52.3%)	18 (51.4%)	
female	46 (46.5%)	12 (54.5%)		41 (47.7%)	17 (48.6%)	
**Smoking history**			0.548			0.979
yes	52 (52.5%)	10 (45.5%)		44 (51.2%)	18 (51.4%)	
no	47 (47.5%)	12 (54.5%)		42 (48.8%)	17 (48.6%)	
**Lymph node metastasis**			**0.004**			**0.000**
yes	41 (41.4%)	2 (9.1%)		40 (46.5%)	3 (8.6%)	
no	58 (58.6%)	20 (90.9%)		46 (53.5%)	32 (91.4%)	
**TNM stage**			**0.027**			**0.010**
I-II	61 (61.6%)	18 (81.8%)		50 (58.1%)	29 (82.9%)	
III-IV	38 (38.4%)	4 (18.2%)		36 (41.9%)	6 (17.1%)	

Bold indicate the expression of RGC32 and N-cadherion was correlated with lymph node metastasis and TNM stage.

**Figure 6:**
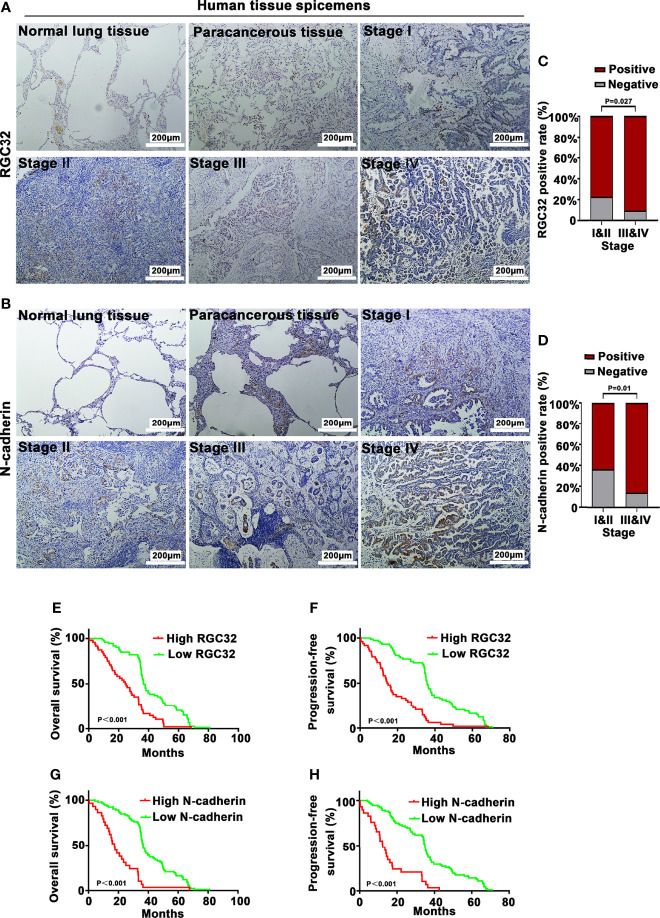
RGC32 and N-cadherin expression and survival analysis of 121 lung adenocarcinoma patients. IHC to detect **(A)** RGC32 and **(B)** N-cadherin in normal lung tissue, paracancerous tissue, and different stages of lung adenocarcinoma. Representative images shown. Magnification ×400. Percentages of **(C)** RGC32 and **(D)** N-cadherin expression in stages I & II and stages III & IV (n = 79 in stages I & II group, n = 42 in stages III & IV group). Kaplan–Meier plots of overall survival and progression free survival of patients with **(E, F)** high and low expression of RGC32, and **(G, H)** high and low expression of N-cadherin in primary lung cancer specimens.

RGC32 and N-cadherin were expressed in normal lung tissues, paracancerous tissues, and LADC tissues, but the highest expression was found in lung cancer tissues (**Figure 6A** for RGC32, **Figure 6B** for N-cadherin). The percentages of RGC32 expression in patients at stages I and II and stages III and IV were 77.2% and 90.5%, respectively (**Figure 6C**), and those of N-cadherin expression were 63.3% and 85.7% (**Figure 6D**), respectively.

In all 121 patients, high RGC32 expression had an adverse effect on the OS (HR = 2.324, 95% CI = 1.512–3.573, P < 0.0001) and PFS (HR = 2.73, 95% CI = 1.74–4.258, P < 0.0001) (**Figures 6E, F**). High N-cadherin expression also had an adverse effect on OS 

## Discussion

In this study, we found that bevacizumab upregulated the expression of RGC32 through activation of the MAPK and PI3K pathways at higher concentration, thereby promoting tumor metastasis and invasion. Simultaneously, anlotinib downregulated the expression of RGC32 by inhibiting the MAPK and PI3K pathways and reversed aberrant biobehaviors of LADC induced by bevacizumab. We suggest that RGC32 is an important molecule involved in bevacizumab resistance.

RGC32 is a cell cycle activation gene that is widely expressed in various human tissues and organs (23–27). We found that it was expressed in many LADC cell lines. Interestingly, the expression of RGC32 in cell lines with EGFR-TKI sensitive mutations (HCC-827, NCI-H1650, and NCI-H3255) was lower than that in cell lines with the EGFR T790M mutation (NCI-H1975). The expression of RGC32 was higher in cells with EGFR-TKI resistant mutations than in those with EGFR-TKI sensitive mutation, which may be worthy of further investigation because of the possible connection between RGC32 and resistance to first-generation EGFR-TKIs.

The role of RGC32 in the occurrence and development of lung cancer has been previously reported, and RGC32 has been shown to participate in EMT (24, 28–30). Recent evidence suggests that RGC32 overexpression promotes the invasion and migration of lung cancer cells by inducing EMT through the NF-κB signaling pathway (30) and that the knockout of RGC32 inhibits the growth, migration, and invasion of lung cancer cells (31).

EMT is an important biological process for epithelial-derived malignant tumor cells to acquire migration and invasion abilities; thus, it is a key step in the progress in cancer development (32). N-cadherin is a mesenchymal cadherin that is closely related to EMT in tumors and is important for metastasis and chemotherapy resistance. Abnormal activation of N-cadherin promotes metastasis and is associated with poor prognosis (33, 34). MMPs are proteolytic enzymes that play an important role in promoting invasion of the extracellular matrix and are also important enzymes in EMT (34, 35). It has been shown that the overexpression of RGC32 in various carcinoma cells upregulates N-cadherin (29, 36) and MMP2 (30, 31) to promote EMT and tumor metastasis (30, 31). Therefore, we selected N-cadherin and MMP2 as EMT-related indicators to study the relationship between RGC32 and EMT. In the present study, the expression of RGC32 was positively correlated with the expression of N-cadherin, which suggests that RGC32 exerts an impact on N-cadherin and participates in EMT in LADC, consistent with previous reports (30, 31). Furthermore, the immunohistochemical results demonstrated that the expression levels of RGC32 and N-cadherin were remarkably related to lymph node metastasis and TNM stage in patients with LADC. In addition, the high protein expression of RGC32 and N-cadherin is an important prognostic factor for poor OS and PFS. These findings support the view that RGC32 and N-cadherin play key roles in the development of LADC and are independent prognostic predictors.

**Figure 7:**
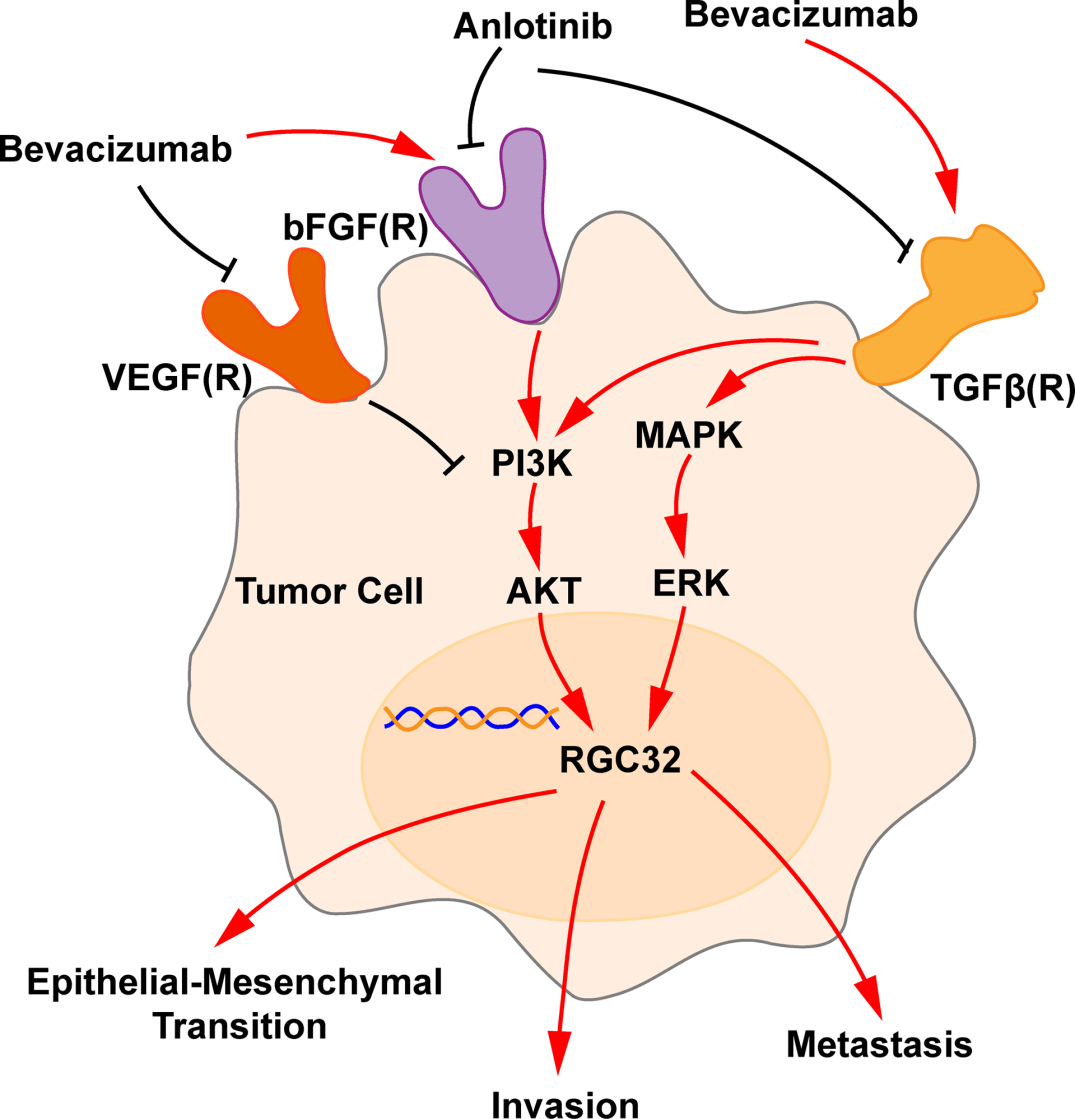
Schematic representation of bevacizumab and anlotinib action in RGC32 signaling pathway. Bevacizumab blocks the VEGF-VEGFR pathway and upregulates the expression of bFGF and TGFβ, which upregulates AKT and ERK, and, accordingly, RGC32 expression, which promotes EMT, invasion, and metastasis. Anlotinib downregulates bFGF(R) axis and TGFβ, thus downregulates RGC32. A black line indicates a blocking action, and a red line indicates a stimulating action.

We also verified that bevacizumab upregulated TGFβ in A549 and H1299 cells at higher concentrations. Similarly, our recently published studies showed that bevacizumab upregulated TGFβ expression in human endothelial cells, mouse retinal microvascular endothelial cells and mouse brain microvascular endothelial cells (17, 37), suggesting that stimulation by higher concentrations of bevacizumab is tangible in several cell lines. Some investigators have shown that FGF upregulates the expression of TGF (38), bevacizumab upregulates bFGF (15), and elevated FGF activates AKT signaling to activate RGC32 (39). TGFβ has also been shown to promote EMT by inducing RGC32 expression *via* the ERK-MAPK pathway (24, 28, 29). Therefore, we suggest that bevacizumab may regulate the expression of RGC32 by upregulating the bFGF-TGF-β, ERK-MAPK and PI3K-AKT pathways, thereby promoting EMT of tumors and metastasis (**Figure 7**). This function may be due to a specific molecular bypass signaling beyond VEGF, since VEGFA was completely neutralized at all concentrations of bevacizumab (**Figure 3G**).

Furthermore, we found that anlotinib downregulated the increased TGFβ by bevacizumab in LADC cells (**Figure 3I**), similar to our results reported in human endothelial cells (17). To verify the inhibitory effect of anlotinib on activated TGF, we applied the HTRF assay that is widely used to test the function of new compounds during drug screening to verify the effect of anlotinib. A549 cells were first activated with TGF-β, and we confirmed that anlotinib indeed inhibits the downstream P-Smad2 mediation of TGFβ (**Figure 3K**), consistent with inhibition of Smad by anlotinib and upregulation of endoglin (another molecule in TGF-β signaling) by bevacizumab as we have previously reported (37). Previous studies have shown that anlotinib inhibits FGFR1-4 (18, 19), whereas bevacizumab can increase the expression of FGFR (15, 16). We believe that anlotinib can downregulate increased RGC32 by inhibiting both FGF/FGFR–AKT and TGFβ/TGFβR-MAPK-ERK pathways (**Figure 7**). To the best of our knowledge, this is the study to prove that anlotinib can inhibit bevacizumab- induced TGFβ/Smad pathway. Moreover, anlotinib downregulated the expression of N-cadherin and MMP2 which are located downstream of RGC32. It also inhibited the migration and invasion of A549 cells and reversed the enhancement of these behaviors induced by bevacizumab. We also found that anlotinib inhibited the elevated protein expression of RGC32, N-cadherin, and MMP2 in tumor tissue induced by induced by bevacizumab in tumor tissues. Due to the abundant evidence connecting N-cadherin and MMP2 with the invasion and metastasis of cancer, the effect of anlotinib identified in the present study indicates that progressive disease can be reduced by resistance to single-targeted anti-angiogenic drugs, as we have recently reported (40).

Bevacizumab has been shown to greatly improve median OS from 10.3 to 12.3 mos (41), although in third-line therapy, none of the previous multi-target TKIs were able to improve the OS for resistant cancer. However, the ALTER0303 trial showed that anlotinib can benefit patients in terms of both PFS and OS (40). Even patients who did not benefit from previous anti-angiogenic therapy can benefit from anlotinib in third or further-line therapy (42) perhaps because of the abolition of LADC cells activation provoked by bevacizumab. To the best of our knowledge, we have further clarified a new mechanism of bevacizumab resistance and have found a novel way to overcome it.

In this study, we defined 100 and 200 mg/mL as high concentrations of bevacizumab. The clinical standard dose of bevacizumab is 7.5–15 mg/kg, approximately converted as follows: depending on the patient’s standard body weight and blood volume (60 kg and 4 L) and regardless of the pharmacokinetics of bevacizumab, the plasma concentration is 60× (7.5–15)/4 mg/L, equivalent to 112.5–225.0 mg/L (i.e., 112.5–225.0 μg/mL). To the best of our knowledge, there has been no canonical study on the concentration of bevacizumab in the lung and other tissues. Plasma concentration of bevacizumab reached 136.3 μg/mL after 21 d of treatment with 15 mg/kg bevacizumab (43). We found that bevacizumab increased TGFβ expression at a concentration of 200 μg/mL (**Figures 3H, I**) when it completely neutralized VEGFA (**Figure 3G**). Noticeably, this level was approximately within the plasma concentration range of clinically routine doses. We therefore believe that the standard dose for the whole body is not necessarily the same as for all lesions and that “excessive concentration” due to tumor heterogeneity and the currently recommended dose of bevacizumab could also provoke the invasive biological action of some malignant cells in some environments, leading to resistance to anti-angiogenic therapy. However, it is impossible and inappropriate to reduce therapeutic doses that may reduce therapeutic efficacy; instead, the purpose of this study was to explore whether improper treatment at “excessive concentrations” of bevacizumab can activate malignant cells, identify the associated mechanisms, and to determine ways to overcome the probable “innate defects” of single VEGF-targeted antiangiogenic drug, thus avoiding the potential clinical risk of “excessive therapy”.

In summary, our data suggest that bevacizumab facilitates tumor metastasis by motivating RGC32 expression through activating the ERK-MAPK and PI3K-AKT pathways in A549 cells. RGC32 and N-cadherin may be indictor of LADC progression and resistance to anti-angiogenic therapy possibly provoked by “excessive therapy” using a single VEGF signaling inhibitor. Anlotinib can reverse resistance to bevacizumab by downregulating the expression of RGC32. This may therefore be a promising therapeutic strategy for overcoming tumor resistance caused by single-targeted anti-angiogenic therapy. Further animal experiments and large-scale clinical trials are needed for verification. Clinical studies of anlotinib combined with chemotherapy for the treatment of first-line lung adenocarcinoma are ongoing (NCT04439890).

However, our study has some limitations in that the molecular mechanisms by which bevacizumab and anlotinib affect the expression or activity of TGFβ have not been examined. Therefore, future studies should focus on elucidating the details of these mechanisms.

## Data Availability Statement

The datasets presented in this study can be found in online repositories. The names of the repository/repositories and accession number(s) can be found in the article/**Supplementary Material**.

## Ethics Statement

The studies involving human participants were reviewed and approved by Medical ethics committee of Tianjin Cancer Hospital. The patients/participants provided their written informed consent to participate in this study. The animal study was reviewed and approved by The Animal Ethical and Welfare Committee of Tianjin Medical University Cancer Institute and Hospital.

## Author Contributions

ZL, TQ, XY, JY, WS, XZ, YJ, SL, JW performed the experiments. ZL produced the figures. ZL and KL designed the study, analyzed the data, and wrote the manuscript. All authors had final approval of the submitted and published versions.

## Funding

This study was supported by grants from Natural Science Foundation of China (81802296 to TQ), Tianjin Municipality Science and Technology Commission Projects (12ZCDZSY15600 to KL), Natural Science Foundation of Tianjin (18JCQNJC82500 to TQ), CSCO (Chinese Society of Clinical Oncology) Special Foundation for Tumor anti-angiogenesis Therapy (Y-X2011-001 to KL), CSCO (Chinese Society of Clinical Oncology) Special Foundation for Tumor anti-angiogenesis Therapy (Y-S2014-011 to JW) and The Science of Technology Development Fund of Tianjin Education Commission for Higher Education (2017KJ201 to TQ).

## Conflict of Interest

Author WS was employed by the company Chia Tai Tianqing Pharmaceutical Group co., LTD.

The remaining authors declare that the research was conducted in the absence of any commercial or financial relationships that could be construed as a potential conflict of interest.

## Publisher’s Note

All claims expressed in this article are solely those of the authors and do not necessarily represent those of their affiliated organizations, or those of the publisher, the editors and the reviewers. Any product that may be evaluated in this article, or claim that may be made by its manufacturer, is not guaranteed or endorsed by the publisher.

## Supplementary Material

Supplementary Figure 1 Bevacizumab promotes H1299 cell migration and invasion. **(A)** Migration and invasion analysis of H1299 cells under different concentrations of bevacizumab. Representative images shown. Magnificent ×200. **(B)** Column chart of migration and invasion cell numbers of H1299 cells for each group. n = 3, Bev, bevacizumab.

Supplementary Figure 2 Effect of bevacizumab on cell proliferation.

Supplementary Figure 3 IC50 of anlotinib in A549 and H1299 cells. **(A)** IC50 in A549 cells. **(B)** IC50 in NCI-H1299 cells.

Supplementary Figure 4 High-dose bevacizumab up-regulated RGC32 expression in H1299 cells. Western blot analysis of RGC32 expression after treatment of bevacizumab. Bev, bevacizumab.

Supplementary Figure 5 RGC32 expression in different lung adenocarcinoma cell lines.

## Abbreviations

LADC, Lung adenocarcinoma; VEGF, Vascular endothelial growth factor; FGFR, Fibroblast growth factor receptor; PDGFR, Platelet-derived growth factor receptor; HTRF, Homogeneous Time Resolved Fluorescence; TKI, Tyrosine kinase inhibitor; DEGs, Differentially expressed genes; EMT, Epithelial-mesenchymal transition; OS, Overall survival; PFS, Progression free survival; TGFβ, Transforming growth factor-β.
