# Supplementary figures and images for: Anlotinib Downregulates RGC32 Which Provoked by Bevacizumab
Source: Front Oncol. 2022 May 18;12:875888. doi: 10.3389/fonc.2022.875888 (PMC9158131; doi:10.3389/fonc.2022.875888)

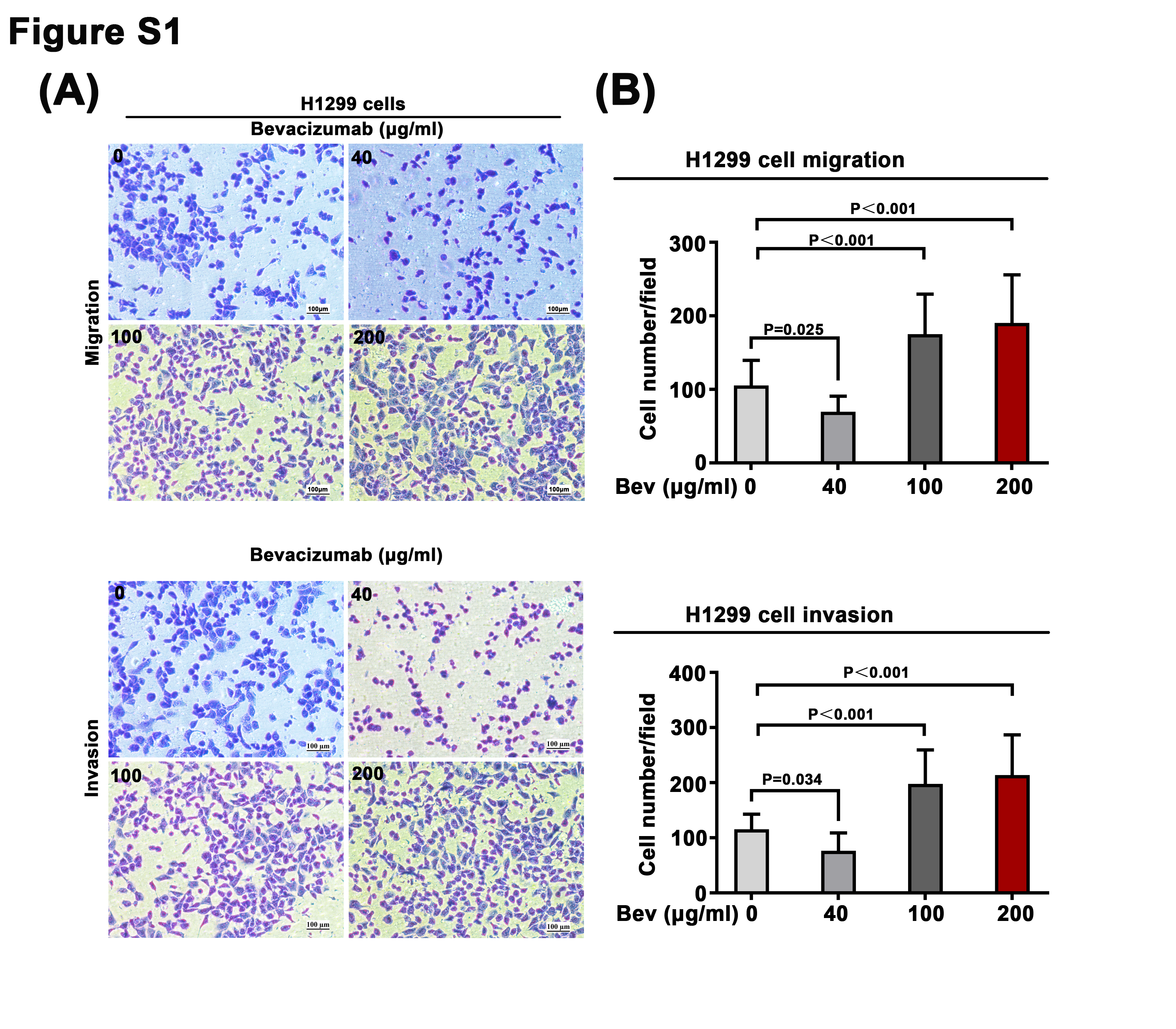

Supplement: Supplementary Figure 1 — Bevacizumab promotes H1299 cell migration and invasion. (A) Migration and invasion analysis of H1299 cells under different concentrations of bevacizumab. Representative images shown. Magnificent ×200. (B) Column chart of migration and invasion cell numbers of H1299 cells for each group. n = 3, Bev, bevacizumab. [file Image_1.tif]

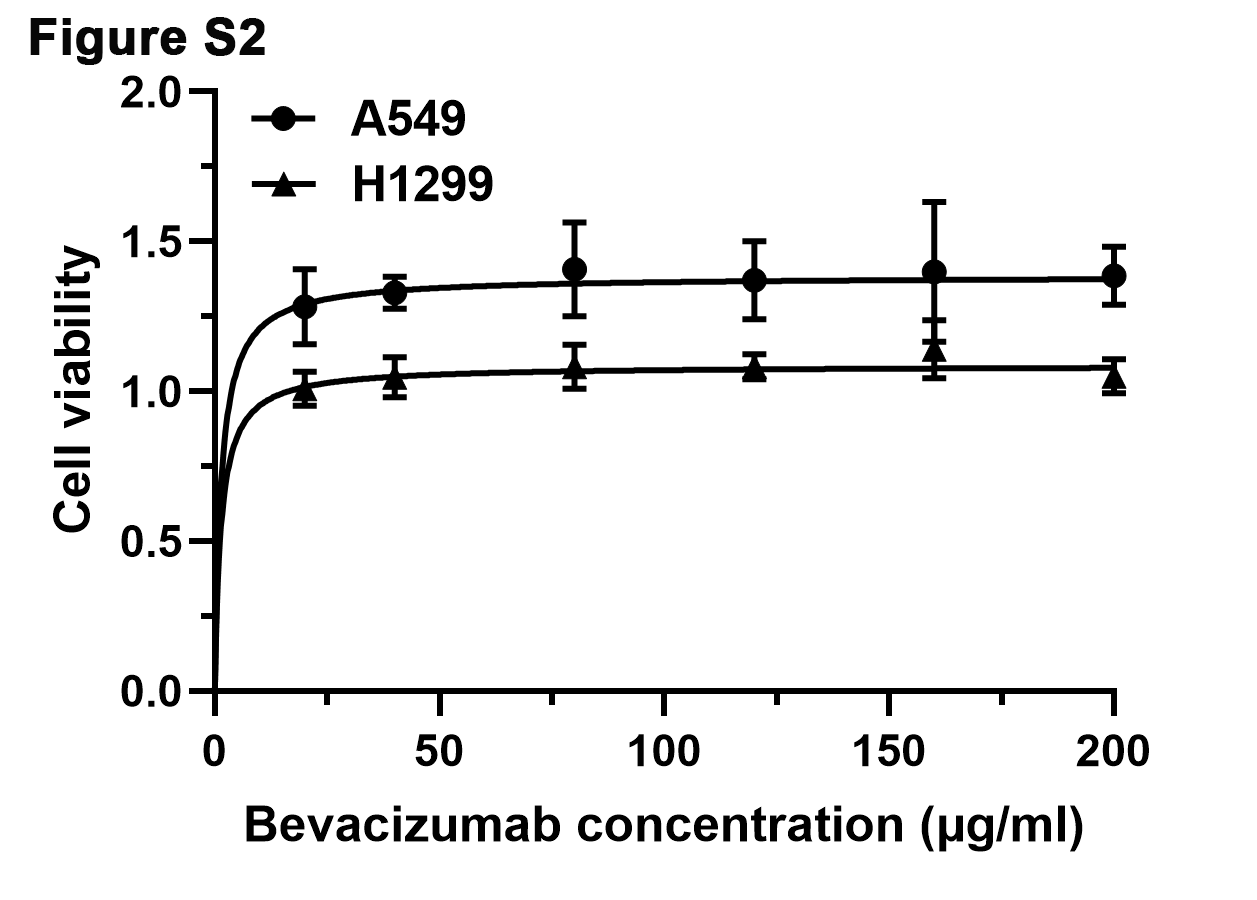

Supplement: Supplementary Figure 2 — Effect of bevacizumab on cell proliferation. [file Image_2.tif]

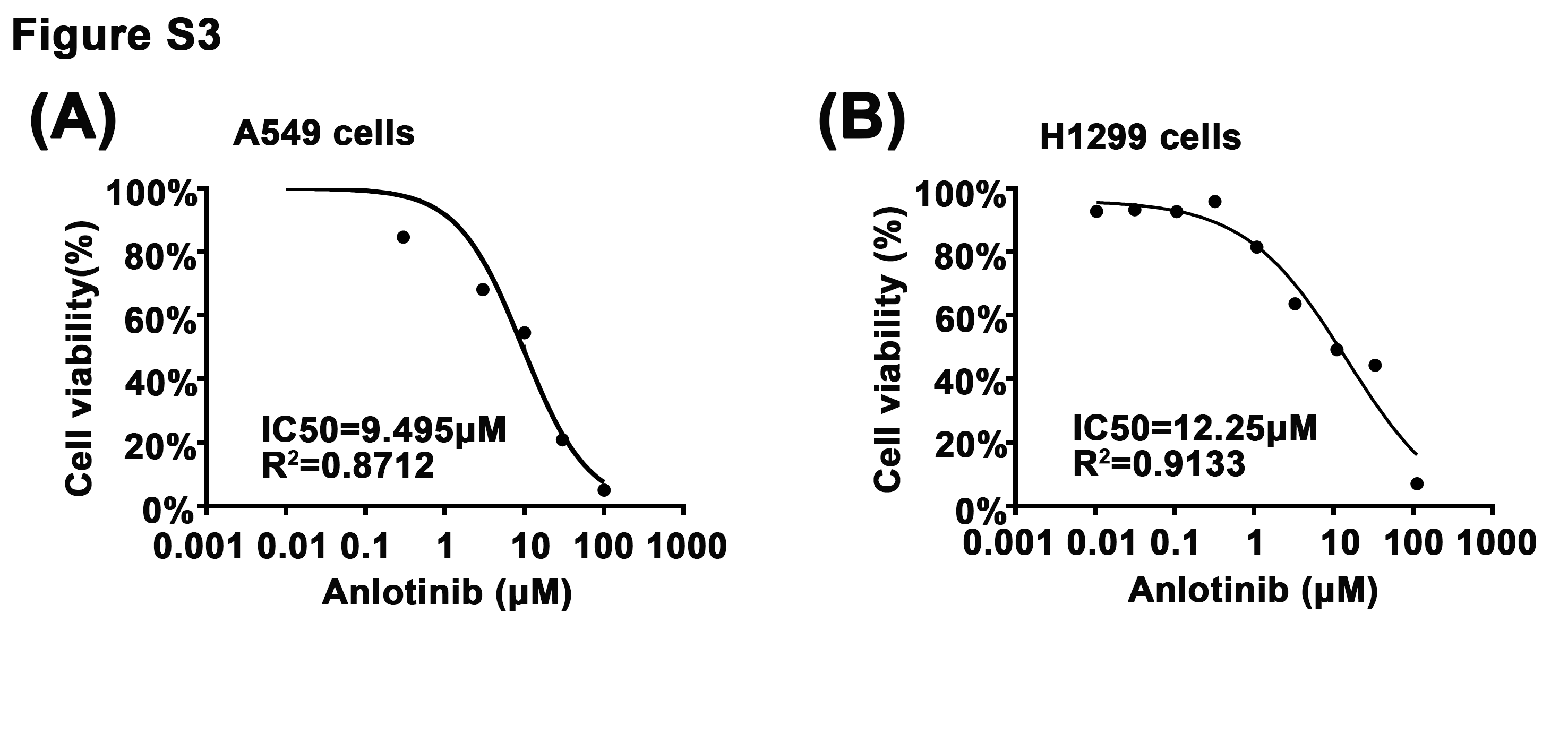

Supplement: Supplementary Figure 3 — IC50 of anlotinib in A549 and H1299 cells. (A) IC50 in A549 cells. (B) IC50 in NCI-H1299 cells. [file Image_3.tif]

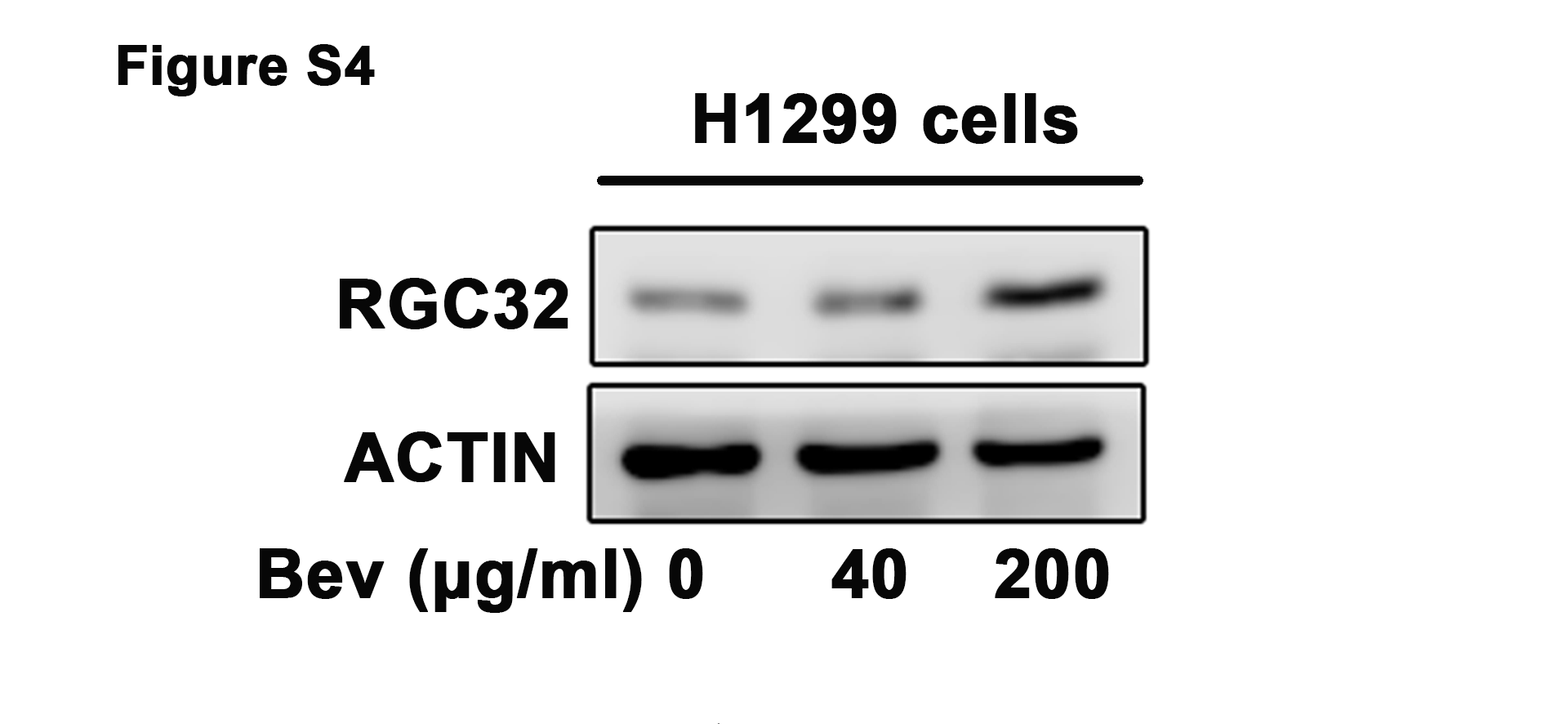

Supplement: Supplementary Figure 4 — High-dose bevacizumab up-regulated RGC32 expression in H1299 cells. Western blot analysis of RGC32 expression after treatment of bevacizumab. Bev, bevacizumab. [file Image_4.tif]

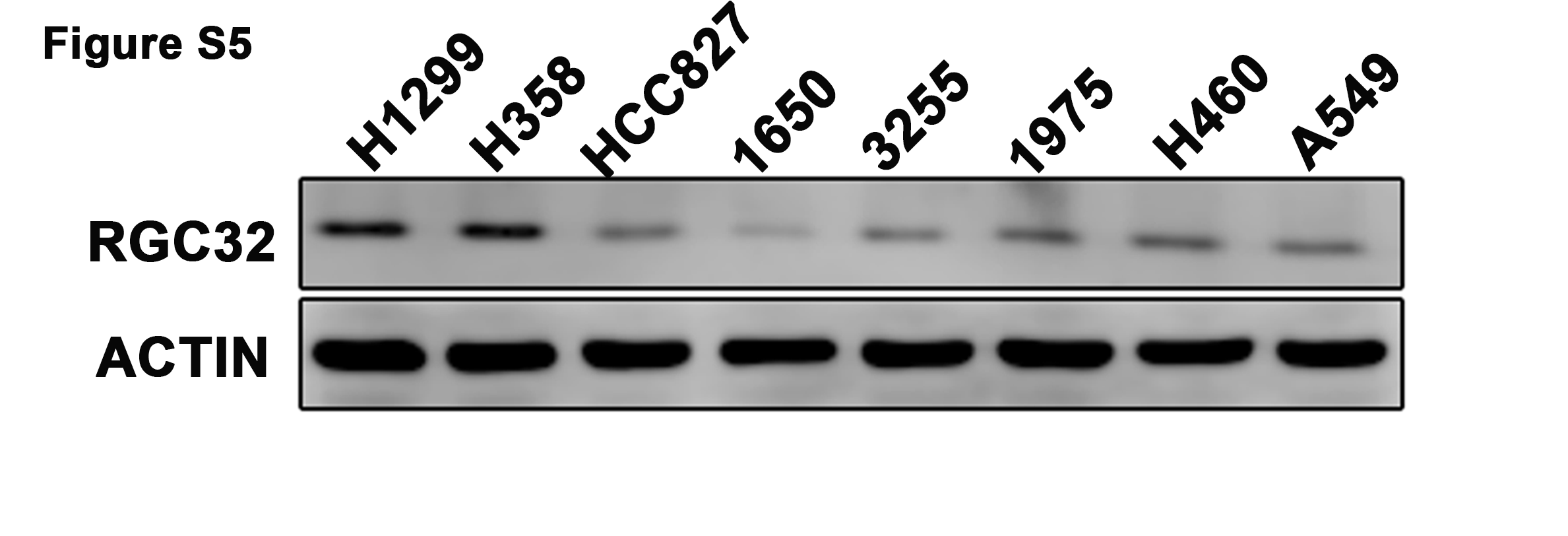

Supplement: Supplementary Figure 5 — RGC32 expression in different lung adenocarcinoma cell lines. [file Image_5.tif]
